# Effect of aspartic acid and glutamate on metabolism and acid stress resistance of *Acetobacter pasteurianus*

**DOI:** 10.1186/s12934-017-0717-6

**Published:** 2017-06-15

**Authors:** Haisong Yin, Renkuan Zhang, Menglei Xia, Xiaolei Bai, Jun Mou, Yu Zheng, Min Wang

**Affiliations:** 1 Key Laboratory of Industrial Fermentation Microbiology, Ministry of Education, College of Biotechnology, Tianjin University of Science & Technology, Tianjin, 300457 People’s Republic of China; 2 School of Bioengineering, Tianjin Modern Vocational Technology College, Tianjin, 300350 People’s Republic of China

**Keywords:** *Acetobacter pasteurianus*, Acid stress resistance enhancement, Aspartic acid, Glutamate, Working mechanisms

## Abstract

**Background:**

Acetic acid bacteria (AAB) are widely applied in food, bioengineering and medicine fields. However, the acid stress at low pH conditions limits acetic acid fermentation efficiency and high concentration of vinegar production with AAB. Therefore, how to enhance resistance ability of the AAB remains as the major challenge. Amino acids play an important role in cell growth and cell survival under severe environment. However, until now the effects of amino acids on acetic fermentation and acid stress resistance of AAB have not been fully studied.

**Results:**

In the present work the effects of amino acids on metabolism and acid stress resistance of *Acetobacter pasteurianus* were investigated. Cell growth, culturable cell counts, acetic acid production, acetic acid production rate and specific production rate of acetic acid of *A. pasteurianus* revealed an increase of 1.04, 5.43, 1.45, 3.30 and 0.79-folds by adding aspartic acid (Asp), and cell growth, culturable cell counts, acetic acid production and acetic acid production rate revealed an increase of 0.51, 0.72, 0.60 and 0.94-folds by adding glutamate (Glu), respectively. For a fully understanding of the biological mechanism, proteomic technology was carried out. The results showed that the strengthening mechanism mainly came from the following four aspects: (1) Enhancing the generation of pentose phosphates and NADPH for the synthesis of nucleic acid, fatty acids and glutathione (GSH) throughout pentose phosphate pathway. And GSH could protect bacteria from low pH, halide, oxidative stress and osmotic stress by maintaining the viability of cells through intracellular redox equilibrium; (2) Reinforcing deamination of amino acids to increase intracellular ammonia concentration to maintain stability of intracellular pH; (3) Enhancing nucleic acid synthesis and reparation of impaired DNA caused by acid stress damage; (4) Promoting unsaturated fatty acids synthesis and lipid transport, which resulted in the improvement of cytomembrane fluidity, stability and integrity.

**Conclusions:**

The present work is the study to show the effectiveness of Asp and Glu on metabolism and acid stress resistance of *A. pasteurianus* as well as their working mechanism. The research results will be helpful for development of nutrient salts, the optimization and regulation of high concentration of cider vinegar production process.

**Electronic supplementary material:**

The online version of this article (doi:10.1186/s12934-017-0717-6) contains supplementary material, which is available to authorized users.

## Background

AAB are gram-negative strict aerobes and have wide distributions in sugar-containing materials or acid materials in nature or alcoholic beverages. They are widely applied in food, bioengineering and medicine fields. They can oxidize ethanol, sugar or sugar alcohol under the existence of oxygen to generate organic acids, such as acetic acid, gluconic acid and indole-3-acetic acid [1–4]. However, the acetic acid which is the main product from AAB is also well known for its cytotoxicity including retardation of growth and product formation [5]. When the acetic acid concentration in the medium increases over 20 g L^−1^, an inhibiting effect begins to be registered. While this effect opposes bacterial growth, when the concentration of acetic acid is over 40 g L^−1^ [6, 7]. The setoxic effects are related to the weak lipophilic nature of the undissociated acid that enables the molecule to cross the cytoplasmic membrane. This diffusion is generally thought to dissipate ion gradients, increase the internal acetic acid concentration, and/or disrupt membrane processes, thereby poisoning the cell [5, 8, 9]. As a result, the cytotoxicity of acetic acid on its microbial producer has become a rate-limiting issue in efforts to further improve the fermentation efficiency of this important natural product and has been the subject of much study.

At present, how to enhance the acid resistance of the producing strain has been extensively studied. The genomes of more than 40 AAB strains from 26 species have been sequenced and released [10–12]. Nakano et al. found that the over-expression of aconitase in *Acetobacter aceti* cells can increase the maximum acid production of AAB by 25% than the original bacteria [13]. Beppu et al. proved that the over-expression of the aldehyde dehydrogenase (ALDH) gene in the original bacteria can increase the acid production by 40% than the original bacteria [14]. Although improving the acid resistance of AAB achieved some results, the acid stress resistance of AAB is a very complex process, and the anti-acid mechanism of AAB is still unclear [4]. Meanwhile, the safety of the obtained mutant remains to be further demonstrated. Therefore, there is an urgent need for a new method which is simple and easy to implement in industry to improve the acid resistance of AAB, promoting acetic acid fermentation efficiency and high concentration of vinegar production.

Amino acids play an important role in cell growth metabolism and survival of cell under severe environment. Callejón et al. reported that AAB consumed amino acid when ethanol was converted to acetic acid [15], and free amino acids in the culture medium were good nitrogen sources of AAB [16, 17]. Sainz et al. reported that amino acid had an important impact on the growth of *Gluconobacter* and *Acetobacter*. The nitrogen requirements for AAB strains are very dependent on the specific strain and the conditions (nitrogen concentration and media) [18]. It is proved that amino acids are important to protect bacteria from environmental stresses. Fulde et al. discovered that *Streptocccus suis* adjusted dynamic equilibrium of intracellular pH by producing NH_3_ and ATP with the consumption of arginine [19]. Tanaka et al. reported that adding lysine to the broth could improve survival of *Vibrio parahaemolyticus*, which indicated that lysine decarboxylase was important for increasing the acid resistance of cells [20]. Senouci-Rezkallah et al. studied effects of glutamate, arginine and lysine on acid tolerance of *Bacillus cereus* ATCC14579 [21], finding that adding these amino acids could maintain dynamic equilibrium of intercellular pH and increase acid stress (pH 4.0) tolerance of cells. Although the reports have found that amino acids play an important role in the production of metabolic energy, reducing equivalents and the enhancement of acid stress resistance of lactic acid bacteria [22]. Until now, the effects of amino acids on acetic fermentation and acid stress resistance of AAB have not been fully studied.

In this study, we aimed to explore the effect of amino acids on enhancing cell growth, acid production and acid stress tolerance of *A. pasteurians*. To gain insights into the working mechanism, proteomic technology was used with the analysis of Kyoto Encyclopedia of Genes and Gnomes (KEGG) data and Cluster of Orthologous Groups (COG) to analyze key enzymes of *A. pasteurians* metabolic pathways changed after Asp and Glu were added. The research results will be helpful for development of nutrient salts, the optimization and regulation of high concentration of cider vinegar production process.

## Methods

### Bacterial strain, growth media and culture conditions

*Acetobacter pasteurianus* AC2005 was purchased from China General Microbiological Culture Collection Center (CGMCC) (Storage No.: CGMCC3089). Solid medium, containing 20 g L^−1^ glucose, 15 g L^−1^ yeast extract, 17 g L^−1^ agar and 20 g L^−1^ CaCO_3_, was sterilized at 121 °C for 20 min. After the medium cooled down to about 60 °C, the absolute ethanol was aseptically added to a final concentration of 28 g L^−1^. Seed culture medium, containing 20 g L^−1^ glucose and 15 g L^−1^ yeast extract, was sterilized at 121 °C for 20 min, and then cooled down to the room temperature. Prior to inoculation, the absolute ethanol was aseptically added to a final concentration of 28 g L^−1^. Batch fermentation medium, containing 20 g L^−1^ glucose and 5 g L^−1^ peptone, was sterilized at 121 °C for 20 min. 0.5 g L^−1^ aspartic acid (Asp), 0.5 g L^−1^ glutamate (Glu) and 0.5 g L^−1^ methionine (Met) were aseptically added, respectively. Prior to inoculation, the absolute ethanol was aseptically added to a final concentration of 56 g L^−1^. The primary seed culture for *A. pasteurianus* was prepared by inoculating cells grown on solid medium into 250 mL Erlenmeyer flasks containing 40 mL seed culture medium and cultured at 30 °C for approximately 27 h (OD610 = 1.38 ± 0.12, middle and later exponential phase) in the rotary shaker (ZWYR-D2402, Shanghai ZHICHENG) at 180 rpm. The secondary seed culture for *A. pasteurianus* was prepared by inoculating the primary seed cells into 500 mL Erlenmeyer flasks containing 100 mL seed culture medium and cultured at 30 °C for approximately 27 h (OD610 = 1.38 ± 0.12, middle and later exponential phase) in the rotary shaker at 180 rpm. The secondary seed prepared for the 1 L fermentors was cultured in four Erlenmeyer flasks each time. After culturing and mixing the seed culture broth, 70 mL of the mixture was transferred to quadruple parallel fermentors with a working volume of 1 L (Multifors Bacteria, INFORS) containing 630 mL of the fermentation medium, which had the same components with the 700 mL fermentation medium, and the total initial fermentation volume was 700 mL. The initial pH of the fermentation medium was not adjusted after sterilization, and the pH and DO was not controlled in the fermentation process. The fermentors were operated at a constant temperature of 30 °C, an agitation rate of 700 rpm and an aeration rate of 0.7 vvm. Biomass and culturable cell counts, acetic and alcohol concentration were measured during the fermentation process. The fermentation was terminated when alcohol concentration was lower than 5 g L^−1^ in the fermentation broth. All fermentation experiments were performed at least twice, to ensure the trends observed were real and reproducible. The data presented in the figures are the average values.

### Proteome analysis

#### Protein preparation

After 16 h culture, the *A. pasteurianus* cell pellets were collected at 10,000×*g* for 10 min at 4 °C. The cell pellets were washed by phosphate buffer saline (PBS) buffer (137 mmol L^−1^ NaCl, 2.7 mmol L^−1^ KCl, 10 mmol L^−1^ Na_2_HPO_4_, 1.76 mmol L^−1^ KH_2_PO_4_, pH 7.4) for three times [23], and then resuspended by PBS. The suspensions was sonicated with a circulation of 40 w for 3 s and pause 5 s until the microbial solutions became totally transparent and then centrifuged at 12,000×*g* for 10 min at 4 °C. The supernatant was mixed well with 4 × volume of chilled acetone and incubated at 20 °C overnight. After centrifugation at 12,000×*g* for 10 min at 4 °C, the supernatant was discarded. The protein sediment was air-dried and kept at −80 °C for further analysis.

#### Protein identification

The acetone precipitated protein was redissolved with 8 mol L^−1^ Urea, and the protein concentration was measured by bicinchoninic acid (BCA) protein assay kit (Product No. 23227, Thermo Scientific). Proteins were collected 150 μg from each sample for sodium dodecyl sulfate–polyacrylamide gel electrophoresis (SDS-PAGE). After that all sample gel strips were conducted by in-gel enzyme digestion, extraction and dryness, they were further analyzed through liquid chromatography–mass spectrometry (LC–MS) (LC:Dionex Ultimate 3000, MS:LTQ Orbitrap Velos Pro, Thermo Scientific).

#### LC–MS data analysis

The MS/MS spectra from each LC–MS/MS run was searched against the NCBI *A. pasteurians* protein database (http://www.ncbi.nlm.nih.gov/). The search algorithm (Sequest HT) in Proteome Discoverer version 1.4 was used for comprehensive protein identification and relative quantification. The search parameters were as follows: one missed cleavage was allowed; carbamidomethylation (C) was set as the fixed modifications; oxidation (M) was set as variable modification; precursor ion mass tolerances were set at 20 ppm for MS acquisition in an Orbitrap mass analyzer; and fragment ion mass tolerances were set at 20 mmu for MS2 spectra acquisition. Peptide spectral matches (PSMs) were validated using the percolator provided by the Proteome Discoverer software based on q-values at a 1% false discovery rate.

#### Function method description

Functional annotations of the proteins were conducted using Blast2GO program against the non-redundant protein database (NR; NCBI). The Cluster of Orthologous Groups (COG) database (http://www.ncbi.nlm.nih.gov/COG/) was used to classify and group the identified proteins [23].

### Analytical method

#### Biomass assay

The fermentation liquor was centrifuged for 10 min at a rate of 6000×*g*. Then the supernatant was eliminated. Subsequently, bacterial cells were taken into a drying oven and dried to constant weight under 105 °C. The dry cells were weighted and the biomass was calculated according to the standard curve.

#### Culturable cell counts assay

Appropriate amount of fermentation broth was collected and step diluted by steriled fresh seed culture medium into an appropriate concentration. Culturable cell counts were measured by plate colony count method [24].

#### Acetic concentration assay

Acid–base titration was applied. Fermentation broth (1 mL) was collected in a 250 mL Erlenmeyer flasks and 15 mL DI water was added. Phenolphthalein was used as the indicator and 0.1 mol L^−1^ sodium hydroxide solution was used to titrate the fermentation broth until its color became pink. Total acid content was measured according to the consumed NaOH.

#### Yield calculation

Calculation formula of yield was seen below:

$${ \text {yield} \,(\%)=\frac {\text{Y}_{\text{acid}}\,(\text{g}\,\text{L}^{-1})}{\text{C}_{\text{ethanol}}\,(\text{g}\,\text{L}^{-1})\,\times\,1.304}}\,\times\,100\%$$

where Y_acid_ present the concentration of acetic acid in fermentation broth (g L^−1^). C_ethanol_ present the concentration of ethanol, and 1.304 present 1 g L^−1^ ethanol completely transformed to obtain 1.304 g L^−1^ acetic acid.

#### Acetic acid production rate calculation

The rate of acetic acid production was estimated from the ratio of the increased acetic acid production versus interval time.

#### Specific production rate of acetic acid calculation

Specific production rate of acetic acid was calculated as described earlier [6].

#### Intracellular ammonia concentration assay

50 mL of bacterium cells was collected after 16 h cultivation and the cell concentration was adjusted to OD = 3.0. The cells of bacteria were washed by 0.2 mol L^−1^ phosphate buffer (pH 7.5) for twice and centrifuged at 10,000×*g* for 5 min at 4 °C. The collected sediment was resuspended in 10 mL same buffer solution, followed by 10 min ice-bath ultrasonic treatment and re-centrifuged at 12,000×*g* for 10 min at 4 °C, the supernatant was collected and intracellular ammonia (as ammonium ions, $\text {NH}^+_{4})$ concentration was assayed according to the instruction of AMMONIA (Rapid) kit (Megazyme Company).

#### Intracellular pH assay

Intracellular pH was assayed as described earlier [25].

### Data processing

Each experiment had 3 parallel tests and data in figures were means of 3 independent tests. Data in graphs were analyzed by Origin 7.5 software.

## Results and discussions

### Determination of key growth factor

Among 16 types of amino acids, it was found that only Asp, Glu and Pro could enhance the cell growth significantly (see Additional file 1: Figure S1). Meanwhile, we found that the content of Asp and Glu in cider was the highest. In addition, it was reported that Glu was the direct biosynthetic precursor for Pro [26], and nutritional studies with isotopic carbon also have provided strong evidence for the interconversion in vivo of Glu and Pro in microorganisms [27, 28]. Thus, we mainly test the effect of Asp and Glu on the production of acetate and acid stress resistance of *A. pasteurians* by proteomic technology with the analysis of KEGG data and COG data.

### Effect of adding Asp and Glu on acetic acid fermentation of *A. pasteurianus*

**Fig. 1:**
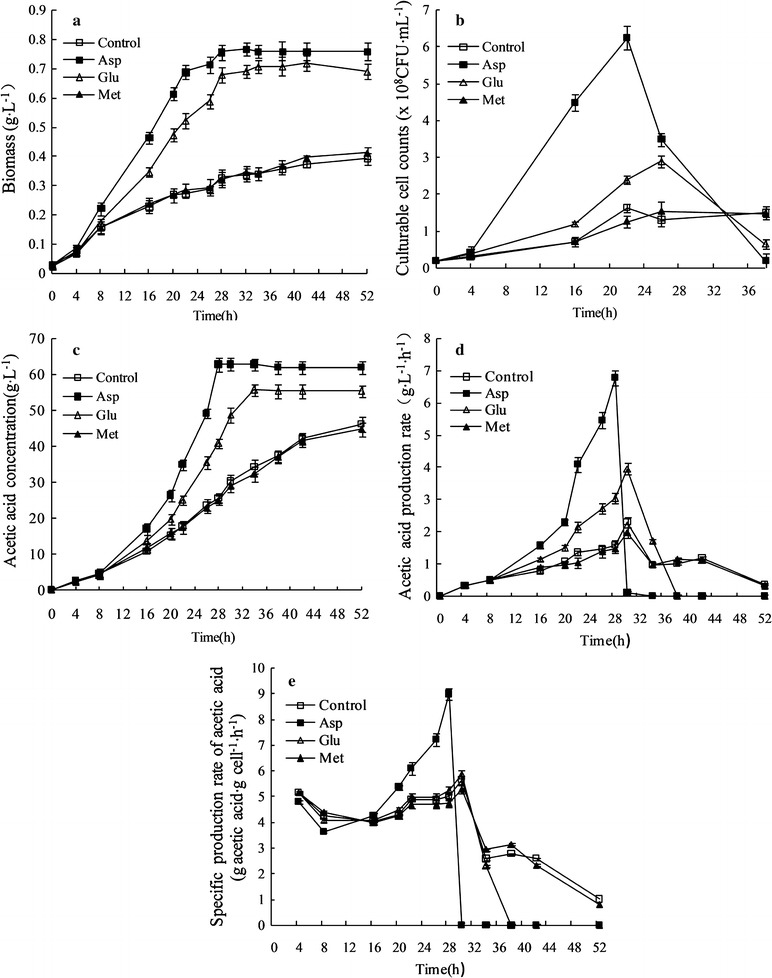
Effects of Asp, Glu and Met on acetic acid accumulation and cell growth during acetic acid batch fermentation of *A. pasteurianus* in 1 L quadruple parallel fermentors. Bacterial growth: **a** Biomass, **b** Culturable cell counts; Acetic acid fermentation: **c** Acetic acid concentration, **d** Acetic acid production rate, **e** Specific production rate of acetic acid. Data represent mean ± standard deviation from three replicates. If *error bars* are not visible they are smaller than the symbol

In this study, the bacteria were cultured in 1 L quadruple parallel fermentors. Results showed that adding Asp and Glu could facilitate the growth of *A. pasteurians* obviously (Fig. 1a, b). After 16 h of fermentation, the biomass reached 0.47 and 0.35 g L^−1^, which had 1.04 and 0.51-folds higher than that of the control group respectively. Culturable cell counts reached 4.5 × 10^8^ and 1.2 × 10^8^ CFU mL^−1^, which had 5.43 and 0.72-folds higher than that of the control group. And the culturable cell counts decreased rapidly in the late stage of acetic acid fermentation, because the death rate of culturable cells was correlated with acetic acid concentration. When the concentration of acetic acid was over 40 g L^−1^, the death rate of culturable cells increased rapidly [6]. However, the addition of Met (control amino acid) showed nearly no effect on biomass and culturable cell counts of *A. pasteurians*.

Additionally, adding Asp and Glu could also facilitate the acid production of *A. pasteurians* obviously (Fig. 1c, d, e). After 28 h of fermentation, acetic acid concentration reached 63 and 41 g L^−1^, which had 1.45 and 0.6-folds higher than that of the control group respectively. Acetic acid production rate reached 6.8 and 3.1 g L^−1^ h^−1^, which had 3.33 and 0.94-folds higher than that of the control group respectively. Specific production rate of acetic acid reached 9.0 g acetic acid g cell^−1^ h^−1^, which had 0.79-folds higher than that of the control group with the addition of Asp, while the effect of adding Glu on the specific production rate of acetic acid was not significant. The yield was highest when the Asp was added, which was 87%. Because of ethanol and acetic acid will be volatilized in the fermentation process, the actual yield was lower than the theoretical yield. However, the addition of Met (control amino acid) showed nearly no effect on acetic acid production, production rate and specific production rate of acetic acid.

The above results showed that adding Asp and Glu could significantly facilitate acetic acid fermentation of *A. pasteurianus*. Especially, adding Asp could significantly improve the fermentation efficiency and increase culturable cell counts. The reason may be that adding Asp could improve key metabolic pathway and acetic acid tolerance of *A. pasteurianus*. The following section, with the help of proteomic technology, will focus on the working mechanism of adding Asp and Glu on metabolism and acid stress resistance of *A. pasteurians*.

### Proteome analysis of *A. pasteurians* cultivated with Asp and Glu adding

#### Functional categorization of differentially expressed proteins of *A. pasteurianus* cultivated with Asp and Glu adding

On the basis of COG analyses, 54 differently expressed proteins classified into 17 COG functional categories were identified with the addition of Asp. Meanwhile, 42 differently expressed proteins classified into 16 COG functional categories were identified with the addition of Glu. Moreover, when Met was added, 54 differently expressed proteins classified into 17 COG functional categories were identified. The analysis data and graphs are shown in the Supporting Information (see Additional file 1: Figures S2–S4).

#### Analyses of differentially expressed proteins related with the growth and acid production of *A. pasteurians*

**Fig. 2:**
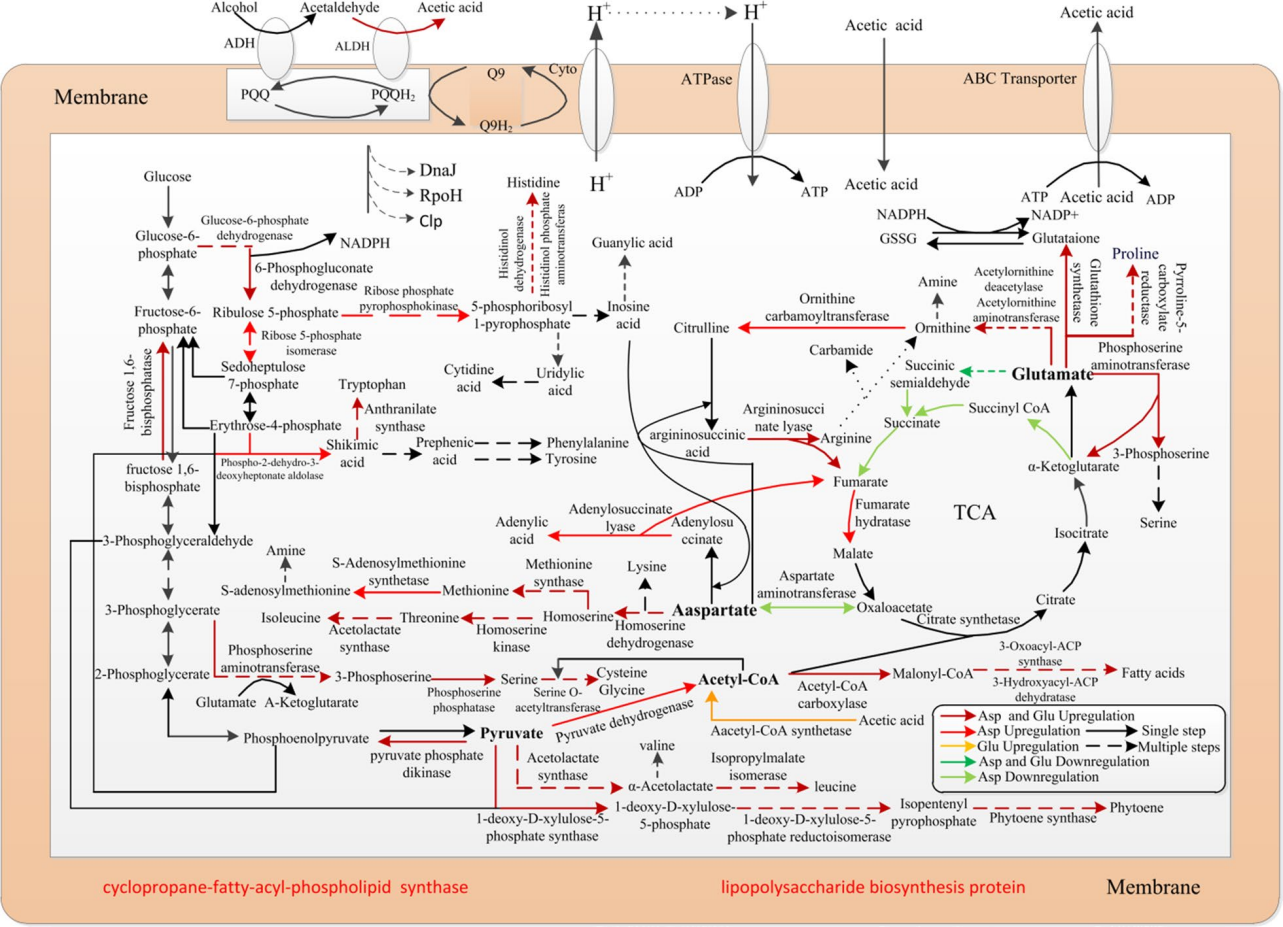
Effects of adding Asp, Glu and Met on the metabolic pathway of *A. pasteurians*

According to proteome analyses, compared with the control group, the key enzymes expressions of *A. pasteurians* metabolic pathways were changed with the addition of Asp and Glu (Table 1; Fig. 2). The ratios of different protein expressions with the addition of Asp Glu and Met to those corresponding controls were shown in Table 1.

**Table 1:** Different expressed proteins (folds more than 1.5 and less than 0.75) with the addition of Asp, Glu and Met

Fuctions	Metabolism passway	Accession	Description	Species	Score	Unique	Change folds		
						Peptides	Asp	glu	met
Carbon metoblism and energy metabolism	PPP	gi|489719443	Ribose5-phosphate isomerase	*A. pasteurians*	28	5	1.70	–	–
		gi|765016869	Glucose-6-phosphate dehydrogenase	*A. pasteurians*	26	4	2.0	1.56	–
		gi|517917674	6-phosphogluconate dehydrogenase	*A. pasteurians*	62	1	1.5	1.5	–
	Gluconeogenesis	gi|489725254	Fructose 1,6-bisphosphatase	*A. pasteurians*	57	2	2.5	2.51	–
		gi|504270024	Pyruvate phosphate dikinase	*A. pasteurians*	718	1	2.17	1.70	–
	TCA circulation	gi|489721425	Pyruvate dehydrogenase E1 subunit alpha	*A. pasteurians*	25	1	1.58	–	–
		gi|371459253	Fumarate hydratase	*A. pasteurians NBRC 101655*	30	6	1.51	–	–
		gi|489725322	Succinate dehydrogenase	*A. pasteurians*	109	15	0.75	–	0.75
		gi|489724258	2-oxoglutarate dehydrogenase subunit E1	*A. pasteurians*	99	1	0.64	–	–
		gi|489724256	Dihydrolipoamide succinyltransferase	*A. pasteurians*	64	6	0.67	–	–
		gi|899754336	Succinate-semialdehyde dehydrogenase	*A. pasteurians*	960	2	0.72	–	–
		gi|899754160	4-aminobutyrate aminotransferase	*A. pasteurians*	161	12	0.20	0.28	
	Oxidation of ethanol	gi|21321317	Aldehyde dehydrogenase	*A. pasteurians*	182	5	1.83	1.91	–
	Peroxidation of acetic acid	gi|517918274	Acetyl-CoA synthetase	*A. pasteurians*	116	2	1.52	2.30	1.74
Metabolism of amino acids	Synthesis of histidine	gi|517918099	Phosphoribosyl-AMP cyclohydrolase	*A. pasteurians*	35	3	1.65	–	–
		gi|440346	Histidinol phosphate aminotransferase	*A. pasteurians*	25	4	1.88	1.56	–
		gi|899753794	Histidinol dehydrogenase	*A. pasteurians*	69	3	1.78	1.51	–
	Synthesis of aromatic amino acids	gi|899753727	Phospho-2-dehydro-3-deoxyheptonate aldolase	*A. pasteurians*	61	11	1.55	–	–
		gi|517920086	Anthranilate synthase	*A. pasteurians*	25	1	14.1	14	7.9
	Synthesis of serine family amino acid	gi|899755975	Phosphoserine aminotransferase	*A. pasteurians*	36	5	1.87	1.60	–
		gi|529246832	Serine *O*-acetyltransferase	*A. pasteurians*	26	2	1.6	–	–
		gi|899754953	Cysteine synthase	*A. pasteurians*	26	4	1.51	–	1.9
	Synthesis of pyruvate family amino acid	gi|899755766	Isopropylmalate isomerase	*A. pasteurians*	68	9	1.52	1.55	–
		gi|899753739	Acetolactate synthase	*A. pasteurians*	130	15	1.51	1.51	2.0
	Synthesis of aspartate family amino acid	gi|489725255	Homoserine dehydrogenase	*A. pasteurians*	163	1	2.35	1.77	–
		gi|489719092	Homoserine kinase	*A. pasteurians*	25	3	1.59	1.78	–
		gi|737380809	Methionine synthase	*A. pasteurians*	39	1	1.54	1.56	–
		gi|648238171	S-Adenosylmethioninesynthetase	*A. pasteurians*	440	24	1.51	–	–
		gi|899755650	Aspartate aminotransferase	*A. pasteurians*	568	27	0.66	–	–
	Synthesis of glutamate family amino acid	gi|502522333	Ornithine carbamoyltransferase	*A. pasteurians*	37	5	1.58	–	–
		gi|528529979	Acetylornithine aminotransferase	*A. pasteurians 386B*	36	8	3.22	1.70	–
		gi|489718470	Acetylornithine deacetylase	*A. pasteurians*	25	2	4.75	3.15	2.09
		gi|489721028	Argininosuccinate lyase	*A. pasteurians*	35	5	1.51	1.52	–
		gi|517918877	Pyrroline-5-carboxylate reductase	*A. pasteurians*	28	1	1.9	1.8	–
		gi|899754127	Amino acid dehydrogenase	*A. pasteurians*	36	13	6.05	–	–
Metabolism of nucleic acid	Metabolism of nucleic acid	gi|899755327	Adenylosuccinate lyase	*A. pasteurians*	75	14	1.51	–	–
		gi|899754646	Ribose phosphate pyrophosphokinase	*A. pasteurians*	76	8	4.8	–	–
		gi|899755733	DNA mismatch repair protein MutS	*A. pasteurians*	25	3	1.82	–	–
		gi|489724275	DNA topoisomerase I	*A. pasteurians*	35	9	1.65	–	–
Self-rescuse and defense of cells	Synthesis of fatty acid	gi|517918905	Acetyl-CoA carboxylase	*A. pasteurians*	27	2	2.34	2.09	2.0
		gi|899755298	3-oxoacyl-ACP synthase	*A. pasteurians*	108	8	1.51	1.52	–
		gi|899756052	3-hydroxyacyl-ACP dehydratase	*A. pasteurians*	33	3	1.67	1.54	0.49
	Composition of cytomembrane	gi|502521557	phytoene synthase	*A. pasteurians*	50	9	2.3	2.40	–
		gi|489718439	1-deoxy-d-xylulose-5-phosphate reductoisomerase	*A. pasteurians*	28	4	1.5	1.5	–
		gi|899755523	1-deoxy-d-xylulose-5-phosphate synthase	*A. pasteurians*	225	1	1.51	–	–
		gi|489721501	Squalene-hopene cyclase	*A. pasteurians*	40	5	5.13	3.96	–
		gi|489718724	Cyclopropane-fatty-acyl-phospholipid synthase	*A. pasteurians*	33	4	1.68	1.68	2.9
		gi|899755773	Lipopolysaccharide biosynthesis protein	*A. pasteurians*	25	1	1.52	1.56	–
		gi|371460759	Outer membrane protein	*A. pasteurians NBRC 101655*	32	6	1.52	–	–
	Synthesis of glutathione	gi|899755744	Glutathione synthetase	*A. pasteurians*	33	4	1.51	1.73	–
	Stress proteins	gi|899755711	Clp protease ATP-binding protein	*A. pasteurians*	69	16	1.57	–	–
		gi|256652539	DNA-directed RNA polymerase Heat shock sigma factor RpoH	*A. pasteurians IFO 3283*-*01*-*42C*	31	5	2.44	2.45	–
		gi|489719702	Molecular chaperone DnaJ	*A. pasteurians*	28	2	5.01	3.95	–

–, 0.75 ‹ Change folds ‹ 1.5

Expressions of proteins related with carbon metabolism and energy metabolism varied greatly with the addition of Asp and Glu. Expressions of pyruvate phosphate dikinase and fructose 1,6-bisphosphatase which were the key enzymes of gluconeogenesis pathway [29] were up-regulated by 2.17, 1.7 and 2.5, 2.51-folds respectively (Table 1). More glucose-6-phosphate which was an important precursor substance of PPP could be synthesized by the gluconeogenesis pathway (Fig. 2). Expressions of Glucose-6-phosphate dehydrogenase and 6-phosphogluconate dehydrogenase which were the key enzymes of PPP were up-regulated by 2.0, 1.56 and 1.5, 1.5-folds respectively, Expressions of Ribose 5-phosphate isomerase (Asp: expressions of proteins were up-regulated with only Asp adding) was up-regulated 1.7-folds (Table 1). However, expressions of the key enzymes in TCA circulation and glycolytic pathway did not increase and even expressions of some proteins down-regulated. Experimental results showed that adding Asp and Glu (especially Asp) could increase expression of the key enzymes in PPP and enhance the metabolic pathway of PPP (Fig. 2), and glucose metabolism of *A. pasteurians* was mainly attribute to PPP. The PPP generated not only pentose phosphates to supply their high rate of nucleic acid synthesis, but also provided NADPH, which was required for both the synthesis of fatty acids and cell survival under stress conditions [30]. The oxidized glutathione (GSSG) was reduced for glutathione (GSH) by NADPH, Meanwhile, the expression of glutathione synthetase was up-regulated (Table 1) and synthesis pathway of GSH was enhanced (Fig. 2). It was speculated that GSH, glutathione synthetase, glutathione reductase (GR) and NADPH composed GSH-dependent reduction system, which played an important role in maintain the viability of cells through keeping intracellular redox equilibrium as well as protecting bacteria from low pH, halide, oxidative stress and osmotic stress [31] and helped the protein repairing and DNA synthesis [32], improving the ability of cell acid-resistant stress [33].

Expressions of proteins related with amino acid anabolism pathway were up-regulated with Asp and Glu (especially Asp) adding (Table 1), accompanied with enhancement of amino acid anabolism pathway (Fig. 2). Expressions of homoserine dehydrogenase, homoserine kinase, methionine synthase and S-Adenosylmethionine synthetase (Asp) which were key enzymes of aspartate-family amino acid metabolism were up-regulated and anabolism of threonine, Met and isoleucine were enhanced (Fig. 2). These provided precursor amino acids for biosyntheses of substances like proteins and facilitated the growth of *A. pasteurians*. Especially, expressions of S-Adenosylmethionine synthetase (Asp) were up-regulated with only Asp adding and the synthesis of S-Adenosylmethionine (SAM) were enhanced, which could improve cell metabolism and participate in biosynthesis and metabolism of intracellular proteins, GSH, nucleic acids, phospholipid and hormones. It was an essential substance for cell normal metabolism [34].

The PPP and amino acid anabolism pathway generated pentose phosphates and SAM to supply high rate of nucleic acid synthesis of *A. pasteurians*. Meanwhile, expressions of the key enzyme of nucleotide synthesis pathway were up-regulated and metabolism pathway of nucleic acid was enhanced with Asp adding (Table 1; Fig. 2). Expression of Ribose phosphate pyrophosphokinase (Asp) and adenylosuccinate lyase was up-regulated by 4.8 and 1.51-folds, which enhanced the synthesis of 5-phosphoribosyl 1-pyrophosphate (PRPP) and adenylic acid (Fig. 2). PRPP was an important precursor of denovo synthesis of purine nucleotide and pyrimidine nucleotide, and participated in salvage synthesis of purine nucleotide and pyrimidine nucleotide [35]. Expressions of DNA repair protein were up-regulated with the addition of Asp. DNA topoisomerase I was an important nuclear endoenzyme and changes topology of DNA, thus participating in all key intranuclear processes, such as DNA replication, transcription, recombination and repairing [36–39]. DNA mismatch repair protein MutS could recognize and bind to mismatched bases, and then bind to MutL dimers to initiate mismatch repair [40].

Experimental results showed that the PPP and amino acid metabolism pathways were enhanced with the addition of Asp and Glu (especially Asp), meanwhile, nucleic acid synthesis and DNA repair was facilitated with the addition of Asp (Fig. 2). These pathways not only generated carbon skeletons, PRPP, nucleotides and various amino acids, which participated in biosynthesis and metabolism of intracellular protein, nucleic acid and phospholipid, but also provided NADPH and SAM, which were required for both the synthesis of fatty acids and GSH. These substances played an important role in maintain the viability of cells and cell survival under stress conditions through keeping intracellular redox equilibrium as well as protecting bacteria from low pH, halide, oxidative stress and osmotic stress and helped the protein repairing and DNA synthesis. Therefore, adding Asp and Glu (especially Asp) could facilitate the growth and survival under stress conditions of *A. pasteurians.* Especially the mass reproduction of *A. pasteurians* with Asp adding increased the acid production of bacteria significantly. Meanwhile, expressions of ALDH which was the key enzyme of ethanol oxidation pathway were up-regulated by 1.83 and 1.91-folds respectively, which enhanced acetaldehyde transferring to more acetic acids and intensified ethanol oxidation pathway to produce more acetic acid and energy.

#### Analysis of differentially expressed proteins related with cell self-rescue and defense

There were significant differences in expressions of proteins related to cytomembrane composition with the addition of Asp and Glu (Table 1). The expression of proteins related to synthesis of fatty acid were up-regulated, Acetyl-CoA carboxylase, 3-oxoacyl-ACP synthase, 3-hydroxyacyl-ACP dehydratase and Squalene-hopene cyclase are the key enzyme of fatty acid synthesis, squalene-hopene cyclase facilitated the synthesis of unsaturated fatty acid, cyclopropane-fatty-acyl-phospholipid synthase increased the supply of cyclopropane fatty acids (CFA) on membrane and involved in a response mechanism of *A. pasteurians* to acid stress [41, 42], meanwhile, the PPP provided NADPH which was required for the synthesis of fatty acids, the synthesis of fatty acid were enhanced, which increased cytomembrane fluidity, stability, and survival of cells under low pH condition, promoted acetic acid transport and enhanced acid tolerance of bacteria [43]. Expressions of 1-deoxy-d-xylulose-5-phosphate synthase, 1-deoxy-d -xylulose-5-phosphate reductoisomerase and phytoene synthase which were key enzymes of phytoene synthesis pathway were up-regulated (Table 1), the phytoene synthesis pathway were enhanced (Fig. 2), and phytoene facilitated lipid transport, metabolism and protected cells from harming of acid stress [44]. The expressions of Lipopolysaccharide biosynthesis protein and outer membrane protein (Asp) which were important ingredients of cytomembrane of gram negative bacteria were up-regulated, Lipopolysaccharide biosynthesis protein promoted the synthesis of lipopolysaccharide and thereby enhanced their protection to cells under acid stress [45, 46]. Outer membrane protein (Asp) participated in formation of cytomembrane, maintained integrity of bacterial outer membrane [47], and helped the molecular exchange of ethanol and acetic acid in periplasm and outside cells [48]. Zhang et al. also identified outer membrane proteins by MALDI–TOF–MS in *A. pasteurianus* HSZ3-21 [49], and previous studies also suggested that outer membrane proteins might be related to the resistance to acetic acid [50].

Expressions of stress proteins like molecular chaperone DnaJ were up-regulated with the addition of Asp and Glu. The cooperation of molecular chaperone DnaJ with DnaK and GrpE was crucial to stabilization of the folding of protein-folding intermediates, protein assembly and disassembly, protein secretion and degradation, which ensured correct protein folding under harsh environments [51]. Andrésbarrao et al. also used proteome analysis and found that the main proteins that play a role in the oxidative fermentation process of *A. pasteurians* LMG1262T were general stress proteins, such as chaperones (GroES, GroEL, DnaK, DnaJ, GrpE) [48]. In addition, *A. pasteurianus* IFO 3283 (former name *A. aceti*) had been shown to increase the mRNA levels of groESL and dnaKJ up to 30 min after the exposure to acetic acid [52, 53]. Expressions of DNA-directed RNA polymerase heat shock sigma factor rpoH increased with the addition of Asp and Glu. RpoH controlled the expression of GroEL, DnaKJ, GrpE and ClpB to different extents [52–54]. In addition, the rpoH disruption mutant of *A. pasteurians* NBRC 3283 became apt to be affected by ethanol and acetic acid [55]. The results indicated the principal and significant role of RpoH in acetic acid fermentation and stressor resistance including acetic acid resistance in *A. pasteurians*. The expression of Clp protease ATP-binding protein was also up-regulated with Asp adding. This protein mainly takes charge of the hydroxylation wrongly folded proteins and is related to acetic acid tolerance [56].

Experimental results showed that, compared with the control group, adding Asp and Glu could promote synthesis of unsaturated fatty acids, enhanced lipid transport and metabolism of cytomembrane, improved cytomembrane fluidity, stability and integrity. The fluidity, permeability of cytomembrane and the key enzyme activities were relate to the resistance to environmental stresses of *A. pasteurians* [57, 58]. The expressions of stress proteins with the addition of Asp and Glu were up-regulated which ensured correct protein folding under harsh environments. Therefore, adding Asp and Glu (especially Asp) could enhance the resistance to environmental stresses of *A. pasteurians* and protected cells from low pH and acid stress.

#### The effect of adding Asp, Glu and Met on intracellular ammonia concentration, intracellular and extracellular pH of *A. pasteurians*

**Fig. 3:**
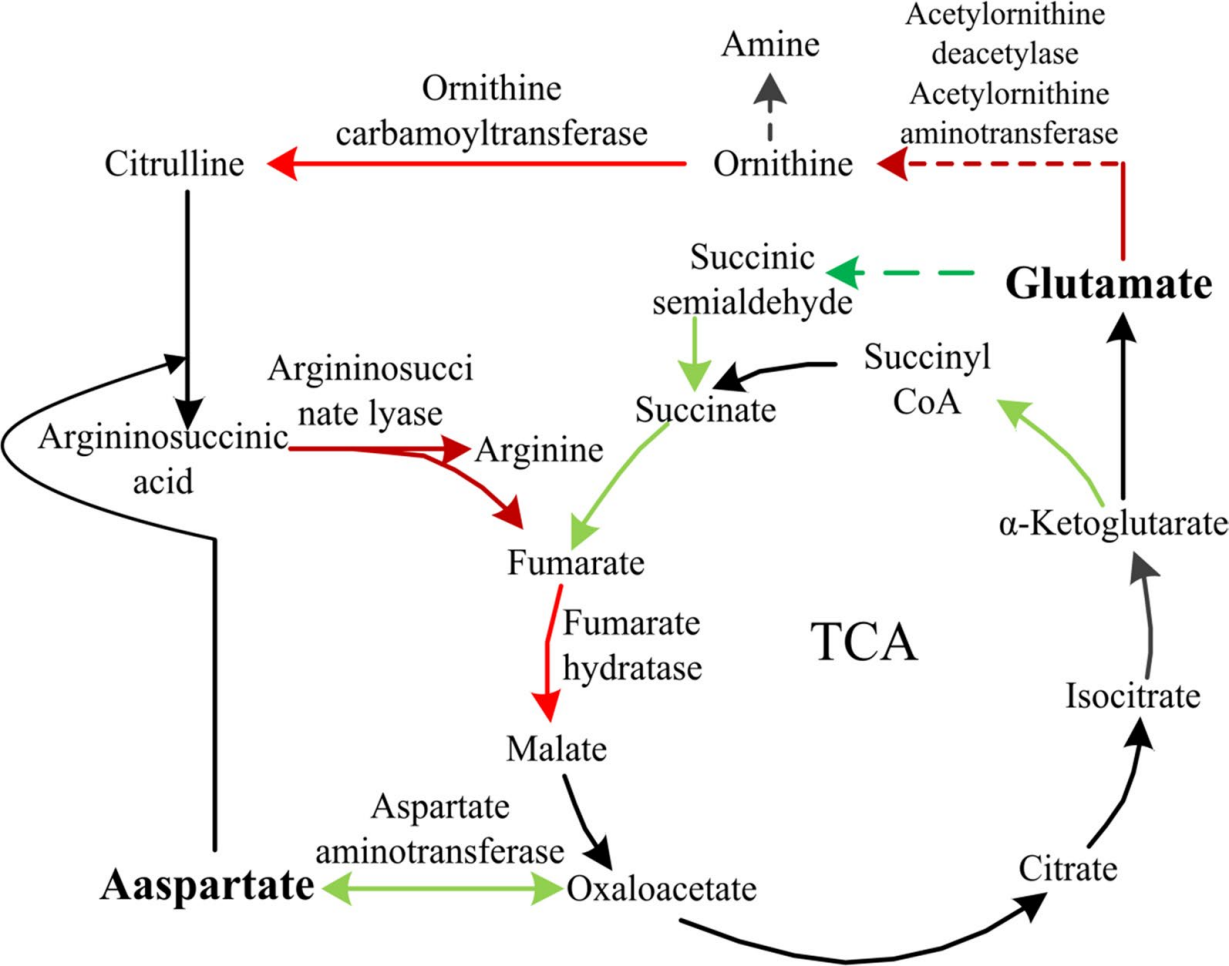
The pathway of ornithine cycle coupling with TCA cycle for *A. pasteurians*

The expressions of acetolactate synthase and isopropylmalate isomerase which were key enzymes of synthesis pathways of leucine and valine were up-regulated by 1.51, 1.51 and 1.52, 1.55-folds respectively with the addition of Asp and Glu. Expressions of homoserine dehydrogenase, homoserine kinase and acetolactate synthase which were key enzymes of synthesis pathway of isoleucine were up-regulated by 2.35, 1.77 and 1.59, 1.78 and 1.51, 1.51-folds respectively. Experimental results showed that the synthesis pathways of leucine, isoleucine and valine were enhanced with the addition of Asp and Glu (Fig. 2). Leucine, isoleucine and valine were all branched-chain amino acids (BCAA). Branched chain amino acids produced NH_3_ during the synthesis process through deamination, which could neutralize acetic acids that entered into cells. It was reported that deamination of BCAA was one of the mechanisms that lactic acid bacteria to maintain intracellular pH stability [59, 60]. Meanwhile, the expressions of ornithine carbamoyltransferase (Asp), acetylornithine aminotransferase, acetylornithine deacetylase and argininosuccinate lyase which were key enzymes for metabolism pathway of arginine were up-regulated significantly (Table 1), the anabolism pathways of ornithine and arginine were enhanced (Fig. 3). Putrescine and NH_3_ were produced to keep the dynamic equilibrium of intracellular pH, and reduce cell injuries which caused by accumulation of intracellular acetic acids [19]. The expressions of amino acid dehydrogenase (Asp) which was the key enzyme for the deamination of amino acids and catalyze deamination of amino acids to produce aminonia were up-regulated by 6.05-folds with the addition of Asp. However, there was no significant change for the expression of the key enzymes which involved in amino acid metabolism with the addition of Met.

**Fig. 4:**
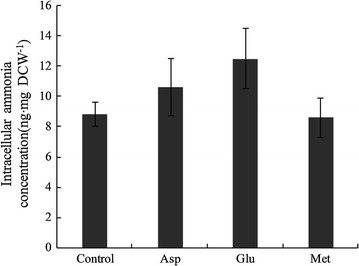
The variations of intracellular ammonia concentration for *A. pasteurians* with the addition of Asp, Glu and Met. Data represent mean ± standard deviation from three replicates. If *error bars* are not visible they are smaller than the symbol

**Fig. 5:**
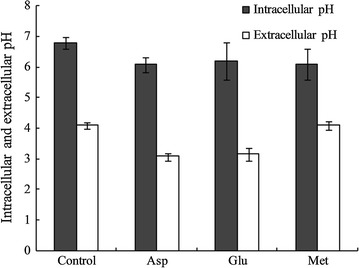
The variations of intracellular and extracellular pH for *A. pasteurians* with the addition of Asp, Glu and Met. Data represent mean ± standard deviation from three replicates. If *error bars* are not visible they are smaller than the symbol

Additionally, the results of intracellular ammonia concentration determination revealed that the intracellular ammonia concentration increased significantly with the addition of Asp and Glu (Fig. 4), which had 0.23 and 0.35-folds higher than that of the control group respectively after 16 h of fermentation. But there was no significant change with the addition of Met. Experimental results showed that, acetic acid could serve as proton carrier for its lipophilic property, which was easy to penetrate the cytomembrane into cells at high concentration level, thus lowered the intracellular pH and influenced normal physiological metabolism of cells [48]. The addition of Asp and Glu could enhance deamination of amino acids and increase the intracellular ammonia concentration, which intensified the ability of neutralizing acetic acids entered in cells to maintain stability of intracellular pH (Fig. 5). Thus, it reduced cell injuries caused by accumulation of acetic acids and facilitated the growth and survival under stress conditions of *A. pasteurians*. Therefore, it was speculated that maintaining intracellular pH stability through deamination of amino acids might be one of the mechanisms to enhance the acid stress tolerance of *A. pasteurians*.

## Conclusions

The present work is the study to show the effectiveness of Asp and Glu on metabolism and acid stress resistance of *A. pasteurianus* as well as their working mechanism. As a result, the addition of Asp and Glu (especially Asp) could improve acid stress resistance of *A. pasteurianus* by enhancing the generation of pentose phosphates and NADPH for the synthesis of nucleic acid, fatty acids and glutathione (GSH) throughout PPP, reinforcing deamination of amino acids to increase intracellular ammonia concentration to maintain stability of intracellular pH, enhancing nucleic acid synthesis and reparation of impaired DNA caused by acid stress damage, promoting unsaturated fatty acids synthesis and lipid transport which resulted in the improvement of cytomembrane fluidity, stability and integrity. The research results will be helpful for development of nutrient salts, the optimization and regulation of high concentration of cider vinegar production process.

## Additional file

**Additional file 1: Figure S1.** Effects of different amino acid on cell growth of *A. Pasteurianus* in Erlenmeyer flasks. **Figure S2.** COG function classification of the identified 54 different expressed proteins with the addition of Asp. **Figure S3.** COG function classification of the identified 42 different expressed proteins with the addition of Glu. **Figure S4.** COG function classification of the identified 54 different expressed proteins with the addition of Met.

## Abbreviations

AAB: acetic acid bacteria
Asp: aspartic acid
Glu: glutamate
Met: methionine
GSH: glutathione
GSSG: oxidized glutathione
ALDH: aldehyde dehydrogenase
KEGG: Kyoto Encyclopedia of Genes and Gnomes
PPP: pentose phosphate pathway
COG: Cluster of Orthologous Groups
PBS: phosphate buffer saline
LC–MS: liquid chromatography–mass spectrometry
SAM: S-Adenosylmethionine
PRPP: 5-phosphoribosyl 1-pyrophosphate
BCAA: branched-chain amino acids
DCW: dry cell weight

## References

[1] Ji A, Gao P (2001). Substrate selectivity of *Gluconobacter oxydans* for production of 2, 5-diketo-d-gluconic acid and synthesis of 2-keto-l-gulonic acid in a multienzyme system. Appl Biochem Biotech..

[2] Merfort M, Herrmann U, Ha SW, Elfari M, Bringer-Meyer S (2006). Modification of the membrane-bound glucose oxidation system in *Gluconobacter oxydans* significantly increases gluconate and 5-keto-d-gluconic acid accumulation. Biotechnol J.

[3] Mamlouk D, Gullo M (2013). Acetic acid bacteria: physiology and carbon sources oxidation. Indian J Microbiol..

[4] Wang B, Shao YC, Chen FS (2015). Overview on mechanisms of acetic acid resistance in acetic acid bacteria. World J Microbiol Biotechnol.

[5] Diezgonzalez F, Russell JB (1997). The ability of *Escherichia coli* O157:H7 to decrease its intracellular pH and resist the toxicity of acetic acid. Microbiology.

[6] Yong SP, Ohtake H, Fukaya M, Okumura H, Kawamura Y (1989). Effects of dissolved oxygen and acetic acid concentrations on acetic acid production in continuous culture of *Acetobacter aceti*. J Ferment Bioeng.

[7] Ory ID, Romero LE, Cantero D (2002). Optimum starting-up protocol of a pilot plant scale acetifier for vinegar production. J Food Eng.

[8] Russell JB (2008). Another explanation for the toxicity of fermentation acids at low pH: anion accumulation versus uncoupling. J Appl Microbiol.

[9] Steiner P, Sauer U (2001). Proteins induced during adaptation of *Acetobacter aceti* to high acetate concentrations. Appl Environ Microbiol.

[10] Azuma Y, Hosoyama A, Matsutani M, Furuya N, Horikawa H, Harada T (2009). Whole-genome analyses reveal genetic instability of *Acetobacter pasteurianus*. Nucleic Acids Res.

[11] Iyer PR, Geib SM, Catchmark J, Kao TH, Tien M (2010). Genome sequence of a cellulose-producing bacterium, *Gluconacetobacter hansenii* ATCC 23769. J Bacteriol.

[12] Ogino H, Azuma Y, Hosoyama A, Nakazawa H, Matsutani M, Hasegawa A (2011). Complete genome sequence of NBRC 3288, a unique cellulose-nonproducing strain of *Gluconacetobacter xylinus* isolated from vinegar. J Bacteriol.

[13] Nakano S, Fukaya M, Horinouchi S (2004). Enhanced expression of aconitase raises acetic acid resistance in *Acetobacter aceti*. FEMS Microbiol Lett.

[14] Beppu T (1993). Genetic organization of *Acetobacter* for acetic acid fermentation. Antonie Van Leeuwenhoek.

[15] Callejón R, Troncoso A, Morales M (2010). Determination of amino acids in grape-derived products: a review. Talanta.

[16] Merrick MJ, Edwards RA (1995). Nitrogen control in bacteria. Microbiol Rev.

[17] Ordóñez JL, Sainz F, Callejón RM, Troncoso AM, Torija MJ, García-Parrilla MC (2015). Impact of gluconic fermentation of strawberry using acetic acid bacteria on amino acids and biogenic amines profile. Food Chem.

[18] Sainz F, Mas A, Torija MJ (2017). Effect of ammonium and amino acids on the growth of selected strains of *Gluconobacter* and *Acetobacter*. Int J Food Microbiol.

[19] Fulde M, Willenborg J, de Greeff A (2011). ArgR is an essential local transcriptional regulator of the arc ABC operon in Streptococcus suis and is crucial for biological fitness in an acidic environment. Microbiology.

[20] Tanaka Y, Kimura B, Takahashi H (2008). Lysine decarboxylase of *Vibrio parahaemolyticu*s: kinetics of transcription and role in acid resistance. J Appl Microbiol.

[21] Senouci-Rezkallah K, Schmitt P, Jobin MP (2011). Amino acids improve acid tolerance and internal pH maintenance in *Bacillus cereus* ATCC14579 strain. Food Microbiol.

[22] Fernández M, Zú iga M (2006). Amino acid catabolic pathways of lactic acid bacteria. Crit Rev Microbiol.

[23] Guo HL, Chen C, Lee DJ, Wang AJ (2014). Coupled carbon, sulfur and nitrogen cycles of mixotrophic growth of *Pseudomonas* sp. C27 under denitrifying sulfide removal conditions. Bioresour Technol.

[24] Ukwo SP, Ezeama CF (2011). Studies on proliferation of acetic acid bacteria during soursop juice fermentaion. Internet J Food Saf..

[25] Breeuwer P, Drocourt J, Rombouts FM (1996). A novel method for continuous determination of the intracellular pH in bacteria with the internally conjugated fluorescent probe 5 (and 6-)-carboxyfluorescein succinimidyl ester. Appl Environ Microb..

[26] Zaprasis A, Bleisteiner M, Kerres A, Hoffmann T, Bremer E (2015). Uptake of amino acids and their metabolic conversion into the compatible solute proline confers osmoprotection to *Bacillus subtilis*. Appl Environ Microb..

[27] Aral B, Kamoun PP (1997). The proline biosynthesis in living organisms. Amino Acids.

[28] Stetten MR (1951). Mechanism of the conversion of ornithine into proline and glutamic acid in vivo. J Biol Chem.

[29] Sakurai K, Arai H, Ishii M, Igarashi Y (2010). Transcriptome response to different carbon sources in *Acetobacter aceti*. Microbiology.

[30] Patra KC, Hay N (2014). The pentose phosphate pathway and cancer. Trends Biochem Sci.

[31] Smimova GV, Oktyabrsky ON (2005). Glutathione in bacteria. Biochem (Moscow).

[32] Jones DP (2006). Redefining oxidative stress. Antioxid Redox Sign.

[33] Zhang J, Fu R, Hugenholtz J (2007). Glutathione protects *Lactococcus lactis* against acid stress. Appl Environ Microb.

[34] Bottiglieri T (2002). S-Adenosyl-l-methionine (SAMe): from the bench to the bedside–molecular basis of a pleiotrophic molecule. Am J Clin Nutr.

[35] Ljungdahl PO, Daignan-Fornier B (2012). Regulation of amino acid, nucleotide, and phosphate metabolism in *Saccharomyces cerevisiae*. Genetics.

[36] Bates A, Maxwell A (2005). DNA topology.

[37] Prommer Y (2012). DNA Topoisomerases and Cancer.

[38] Tuduri S, Crabbé L, Conti C, Tourrière H, Holtgreve-Grez H, Jauch A, Pantesco V, de Vos J, Theillet C, Thomas A, Pommier Y, Tazi J, Coquelle A, Pasero P (2009). Topoisomerase 1 suppresses replication stress and genomic instability by preventing interference between replication and transcription. Nat Cell Biol.

[39] McClendon AK, Rodriguez AC, Osheroff N (2005). Human topoisomerase IIα rapidly relaxes positively supercoiled DNA: implications for enzyme action ahead of replication forks. J Biol Chem.

[40] Kültz D (2005). Molecular and evolutionary basis of the cellular stress response. Annu Rev Physiol.

[41] Chang YY, Cronan JE (1999). Membrane cyclopropane fatty acid content is a major factor in acid resistance of *Escherichia coli*. Mol Microbiol.

[42] Wang WJ, Rasmussen T, Harding AJ (2012). Salt bridges regulate both dimer formation and monomeric flexibility in HdeB and may have a role in periplasmic chaperone function. J Mol Biol.

[43] Sahm H, Rohmer M, Bringer-Meyer S (1993). Biochemistry and physiology of hopanoids in bacteria. Adv Microb Physiol.

[44] Wu JL, Wu QP, Zhang JM, Mo SP, Bai JL (2013). Advances on microbial biosynthesis and fermentation production of lycopene. Food Sci.

[45] Moonmangmee S, Toyama H, Adachi O (2002). Purification and characterization of a novel polysaccharide involved in the pellicle produced by a *thermotolerant Acetobacter* strain[J]. Biosci Biotechnol Biochem.

[46] Kanchanarach W, Theeragool G, Inoue T (2010). Acetic acid fermentation of *Acetobacter pasteurianus*: relationship between acetic acid resistance and pellicle polysaccharide formation. Biosci Biotechnol Biochem.

[47] Weiser JN, Gotschlich EC (1991). Outer membrane protein A (OmpA) contributes to serum resistance and pathogenicity of *Escherichia coli* k-1. Infect Immun.

[48] Andrésbarrao C, Saad MM, Chappuis ML, Boffa M, Perret X (2012). Proteome analysis of *Acetobacter pasteurianus* during acetic acid fermentation. J Proteomics.

[49] Zhang Z, Ma H, Yang Y, Dai L, Chen K (2015). Protein profile of *Acetobacter pasteurianus* HSZ3-21. Curr Microbiol.

[50] Matsutani M, Hirakawa H, Saichana N (2012). Genome-wide phylogenetic analysis of differences in thermotolerance among closely related *Acetobacter pasteurianus* strains. Microbiology.

[51] Gething MJ, Sambrook J (1992). Protein folding in the cell. Nature.

[52] Okamoto-Kainuma A, Wang Y, Ishikawa M (2004). Cloning and characterization of the dnaKJ operon in *Acetobacter aceti*. J Biosci Bioeng.

[53] Okamoto-Kainuma A, Wang Y, Kadono S (2002). Cloning and characterization of groESL operon in *Acetobacter aceti*. J Biosci Bioeng.

[54] Susin MF, Baldini RL, Gueiros-Filho F, Gomes SL (2006). GroES/GroEL and DnaK/DnaJ have distinct roles in stress responses and during cell cycle progression in *Caulobacter crescentus*. J Bacteriol.

[55] Okamotokainuma A, Ishikawa M, Nakamura H, Fukazawa S, Tanaka N (2011). Characterization of rpoH in *Acetobacter pasteurianus* NBRC 3283. J Biosci Bioeng.

[56] Liu YP, Tang HZ, Lin ZL, Xu P (2015). Mechanisms of acid tolerance in bacteria and prospects in biotechnology and bioremediation. Biotechnol Adv.

[57] Boyd DA, Cvitkovitch DG, Beleiweis AS (2000). Defects in d-alanyl-lipoteichoic acid synthesis in streptococcus mutans results in acid sensitivity. J Bacteriol.

[58] Fozo EM, Quivey RG (2004). Shifts in the membrane fatty acid profile of *Streptococcus mutans enhance survival* in acidic environments. Appl Environ Microbiol.

[59] Sánchez B, Champomier-Vergès MC, Collado MC (2007). Low-pH adaptation and the acid tolerance response of *Bifidobacterium longum* biotype longum. Appl Environ Microb..

[60] Len AC, Harty DW, Jacques NA (2004). Proteome analysis of Streptococcus mutans metabolic phenotype during acid tolerance. Microbiology.

