# Supplementary material for: Effect of aspartic acid and glutamate on metabolism and acid stress resistance of Acetobacter pasteurianus
Source: Microb Cell Fact. 2017 Jun 15;16:109. doi: 10.1186/s12934-017-0717-6 (PMC5472864; doi:10.1186/s12934-017-0717-6)
Supplement: Supplementary file 1 — Additional file 1: Figure S1. Effects of different amino acid on cell growth of A. Pasteurianus in Erlenmeyer flasks. Figure S2. COG function classification of the identified 54 different expressed proteins with the addition of Asp. Figure S3. COG function classification of the identified 42 different expressed proteins with the addition of Glu. Figure S4. COG function classification of the identified 54 different expressed proteins with the addition of Met. [file 12934_2017_717_MOESM1_ESM.docx]

**Additional files**


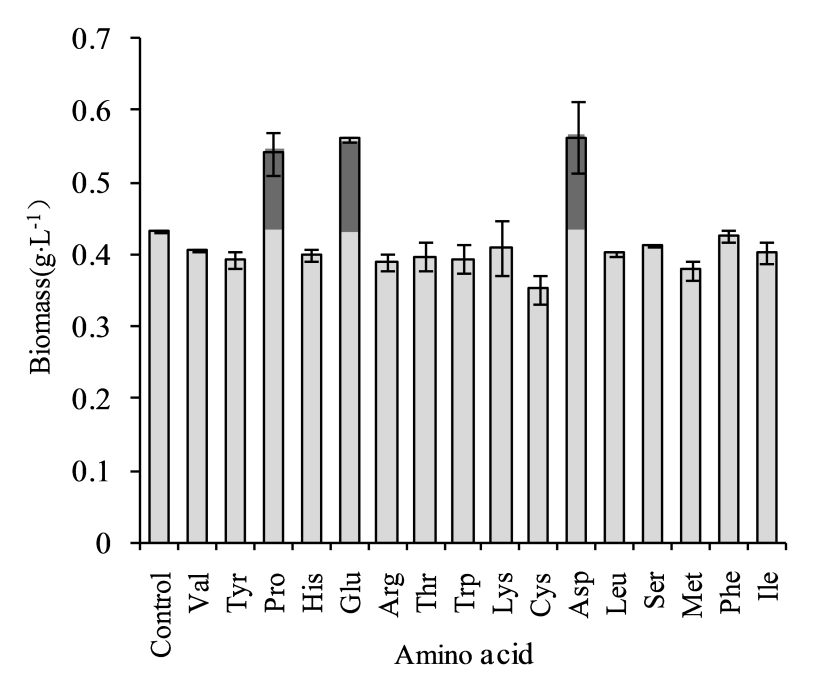


Fig. 1 Effects of different amino acid on cell growth of *A. Pasteurianus* in Erlenmeyer flasks

Fig.2 COG function classification of the identified 54 different expressed proteins with the addition of Asp

Fig.3 COG function classification of the identified 42 different expressed proteins with the addition of Glu

Fig.4 COG function classification of the identified 54 different expressed proteins with the addition of Met
